# Personal Development of Adolescent Peer Leaders of a Whole‐of‐School Physical Activity Programme: Expectations Versus Outcomes

**DOI:** 10.1111/josh.70216

**Published:** 2026-09-10

**Authors:** Kathleen McNally, Elaine Murtagh, Kwok Ng, Caera Grady, Catherine B. Woods

**Affiliations:** ^1^ Physical Activity for Health Research Centre Health Research Institute, University of Limerick Limerick Ireland; ^2^ Department of Physical Education and Sport Sciences University of Limerick Limerick Ireland; ^3^ Institute of Sports Science and Innovation, Lithuanian Sports University Kaunas Lithuania; ^4^ Department of Teacher Education, Turku Institute for Advanced Studies, University of Turku Rauma Finland

**Keywords:** adolescent, expectations, peer leader, physical activity, school

## Abstract

**Background:**

The Active School Flag (ASF) programme for second‐level schools in Ireland is a whole‐of‐school physical activity initiative involving adolescents as peer leaders. This study explores how the peer leadership of this programme influenced adolescents' skills, competencies, perceived health, and school experience. Based on situated expectancy‐value theory (SEVT), we examined how outcomes met peer leaders' expectations as this can influence motivation for their peer leader role.

**Methods:**

Data from peer leaders' focus groups and teacher interviews conducted at beginning and end of academic year were analyzed using reflexive thematic analysis. Informed by SEVT, results were synthesized to identify how expectations compared to experience.

**Results:**

The ASF class fulfilled peer leader expectations, providing a comfortable learning environment. Navigation of implementation challenges resulted in unexpected personal growth opportunities including developing problem‐solving skills. While at the outset peer leaders expected ASF would become more recognizable among the school community, this was unfulfilled.

**Implications for School Health Policy, Practice, and Equity:**

School‐based programmes need to resource and support peer leaders when undertaking challenging tasks. School management should formally recognize peer leaders.

**Conclusions:**

SEVT extends understanding of ways to encourage and sustain peer leaders' programme involvement. This study highlights added‐value and complexities of student‐led initiatives.

## 1 Introduction

Globally, many school‐going adolescents are failing to achieve physical activity (PA) guidelines [1]. To overcome physical inactivity, recommendations suggest education settings apply a whole‐of‐school (WS) approach [2]. The Active School Flag (ASF) programme (www.activeschoolflag.ie) for second level schools in Ireland uses a WS approach [3] to provide adolescents with PA opportunities [4]. Prior to ASF implementation schools must achieve “readiness to engage” criteria. Student voice [5] and leadership [6] are central to ASF as a class of adolescent peer leaders implement ASF with the support of teachers and school management. Peer‐led strategies are commonly used in PA interventions involving adolescents [7, 8, 9], but research is required to explore how they influence programme implementers in addition to receivers [10, 11].

ASF peer leaders are in the fourth year of the Irish second level education that consists of a 1‐year programme called Transition Year (TY) [12]. TY focuses on social and personal development and is typically offered as an optional year to adolescents aged 15–16 years. Schools have autonomy to design their own TY programme within guidelines [13] that addresses students' needs, and ASF is a module that can be offered.

ASF peer leaders' tasks involve planning, implementing, and evaluating PA opportunities to their WS community with the support of ASF resources (Table 1). Research has explored the effects of PA interventions on peer leaders [14], however the impact of peer‐leading the ASF has not yet been examined and recommendations to build on this literature has been outlined [11, 15]. Although peer leaders facilitated ASF implementation, there was a lack of engagement and buy‐in among some leaders [15]. Furthermore, TY students in general were found to lack motivation resulting in unwillingness to work and/or school absence [16]. To ensure successful ASF implementation, peer leaders require support with motivation and engagement.

**TABLE 1:** Description of ASF resources and peer leader tasks.

ASF resources		Peer leader tasks	
ASF class	Dedicated class for ASF embedded in peer leaders school timetable. Typically, one/two classes a week, ranging between 40 and 60 min. Provides opportunity to plan and implement peer leader tasks.	Create school ASF action plan	Peer leaders consider information and ideas provided by general student body in the ASF whole‐of‐school survey report to inform their school ASF action plan.
ASF coordinator and staff committee	Typically, one/two teachers, known as ASF coordinators facilitate the ASF timetabled class and support peer leaders. A group of school staff is identified to support implementation.	Present action plan to school management	Peer leaders meet with school leadership team to discuss ASF action plan.
ASF whole‐of‐school survey	Annual survey that all school members are invited to participate in. Survey questions include students PA behaviors and opinions (e.g., active travel, lunch time PA participation).	Delivery of > 2 whole‐of‐school events	Peer leaders plan, promote, and organize the whole‐of‐school PA events for their school. Every student and staff member are invited, encouraged, and facilitated to participate.
Shared leadership training	A programme used in ASF class empowering peer leaders to take on roles, responsibilities, and leadership positions. To share the workload students are assigned roles within teams for example walkway leaders, media and design, junior/senior class liaison.	Create/promote the ASF walkway	Peer leaders are involved in the planning, measuring, and mapping of a signposted ASF walkway route. The walkway can be used by school members to support physical education curriculum and learning on the move.
External ASF survey report	Peer leaders receive a survey report from an external research team containing data from their school's ASF whole‐of‐school survey. Survey results are presented graphically, table format, and open responses. Peer leaders interpret the data and use it to create school ASF action plan.	Organize ASF active school week	Active school week is an annual national initiative. Peer leaders plan and implement a week of activities to get school members active.
Class ePortfolio	Peer leaders discuss and reflect on the whole‐of‐school event(s) afterwards using the ePortfolio. The ePortfolio contains a review that is submitted as part of ASF accreditation process.	Establish a PA information hub and showcase ASF achievements	Peer leaders promote ASF and PA information through the ‘Did you Know?’ campaign, that can be implemented weekly, fortnightly, monthly, or specific times during the year. A showcase video of ASF activities is also produced at the end of the year highlighting work completed.
Access to ASF school network	Peer leaders engage with students involved in ASF in external schools sharing ideas and experiences.	Hand over plan to students who will be peer leaders next year	Current peer leaders meet with new group of students in the ASF class to discuss work completed and share insights/advice.
Criteria to guide work	Schools must achieve specific requirements outlined in the criteria to progress to each stage of the programme.		
Promotional materials	Peer leaders receive branded ASF resources for example whistles, badges.		
Review of ASF action planning	At end of the academic year peer leaders review their work completed and reflect on the changes they have brought about in their school.		

Abbreviations: ASF = Active School Flag; PA = physical activity; TY = transition year.

Expectancy‐value theory of motivation [17] proposes that interactions between expectations of success and the subjective value beliefs for succeeding determine motivation to complete tasks in educational settings [18]. Situated expectancy‐value theory (SEVT) [19] builds on this, emphasizing that beliefs are situation specific. Expectancy‐value theory was used to explore adolescents' engagement in physical education contexts [20, 21], providing support for its use in this study. Processes influencing motivation of peer leaders of a school‐based PA programme, particularly through a lens like SEVT remain underexplored. Furthermore, it is unclear how these motivational processes influence peer leaders' outcomes.

The primary aim was to explore how peer leadership of a WS PA programme influenced adolescents' skills, competencies, perceived health outcomes and behaviors, and school experience. To inform strategies to support adolescent peer leaders' motivation in peer‐led interventions, the secondary aim was to assess through an SEVT lens the extent to which the primary aim outcomes addressed peer leaders' expectations.

## 2 Methods

### 2.1 Participants

Schools selected were in the second stage of ASF. KM and CW explained the study to ASF coordinators at a standing online ASF training meeting. Following this ASF coordinators received a formal email invitation asking them to participate in an interview and to nominate three to eight peer leaders to complete focus groups.

### 2.2 Instrumentation

Semi‐structured focus group scripts (File SA) were informed by focus group guides from evaluation of a peer‐led intervention [22], models informing ASF [5, 23], consultations with ASF programme developers, and were designed to elicit responses related to expectancy and value constructs of SEVT. The interview script was developed by adapting the focus group script to explore the ASF coordinators' perception of the peer leaders' experiences and outcomes.

### 2.3 Procedure

Peer leader focus groups were completed at the beginning and end of the 2022–2023 academic year to understand their expectations and how the programme addressed their expectations. A moderator took notes and presented discussion summaries. One‐to‐one interviews with ASF coordinators occurred at the end of the academic year. Data collection occurred separately for each school either online or in‐person. Data were collected by KM and CG, both white Irish, female, postgraduate students with previous qualitative experience. Average duration of focus groups and interviews was 44 and 47 min, respectively.

### 2.4 Data Analysis

Focus groups and interviews were audio‐recorded and transcribed verbatim. The following outlines the two analysis phases.

#### 2.4.1 First Analysis Phase

Reflexive thematic analyses [24] were conducted independently for peer leaders focus groups at (1) beginning, (2) end of academic year, and (3) ASF coordinator interviews. A collaborative coding approach was used to generate initial codes. A coding team consisting of KM and three researchers (*n* = 1 male, *n* = 2 female, undergraduate and postgraduate students) that were not involved in planning or data collection, providing detached perspectives. For each component of the primary aim KM and one other coding team member independently, inductively coded the same transcript. This ensured KM had a richer and comprehensive analysis of the data rather than establishing consensus [24]. KM compiled codes into a list of level one codes and proceeded to code the remaining transcripts, maintaining an open iterative coding process. Subsequently, level one codes with a pattern of shared meaning were grouped together to create level two codes, and finally grouped to level three codes. This resulted in preliminary themes and sub‐themes, in which all authors revised and defined the names through re‐engagement with data and codes.

#### 2.4.2 Second Analysis Phase

Preliminary themes and sub‐themes from the first analysis phase were synthesized deductively using an SEVT lens. Outcomes (how ASF influenced peer leaders) reported by ASF coordinators and peer leaders at the end of academic year were collated and mapped to the peer leaders' programme expectations from the beginning of year. Authors examined preliminary themes and sub‐themes and assessed whether there were congruence or discrepancies in descriptions and quotes between the start (expectations) and end (outcomes) of the year. This informed themes and sub‐themes that were categorized based on the expectancy for success component of the SEVT as follows: (i) fulfilled (outcome peer leaders expected at the beginning of year was achieved, i.e., preliminary themes and sub‐themes with congruence at beginning and end of the year), (ii) unfulfilled (outcome peer leaders expected at beginning of year was not achieved, i.e., preliminary themes and sub‐themes only present at start of the year), and (iii) unexpected (outcome achieved that peer leaders did not expect at beginning of year, i.e., preliminary themes and sub‐themes only present at end of the year). For example, sub‐theme “*Boosts confidence*” (fulfilled outcome) was derived by alignment between peer leader data at the beginning of the year (preliminary sub‐theme “Develop confidence in themselves”) and ASF coordinator data at the end of the year (preliminary sub‐theme “ASF boosted peer leaders' confidence”) (File SB). Disagreements among authors were addressed through discussions to reach consensus.

## 3 Results

### 3.1 Participant Characteristics

Of the 12 schools in the second stage of ASF, nine were recruited (Table 2). Focus groups (*n* = 17) and interviews (*n* = 6) were completed among mixed, boys‐only, girls‐only, Delivers Equality of Opportunity in Schools (DEIS) [25] and non‐DEIS schools. DEIS provides a pathway through education to better opportunities for students in communities at risk of disadvantage and social exclusion. One school had two ASF coordinators participate in the interview at the same time. Twenty‐four peer leaders completed the focus groups at both time points. Themes and sub‐themes derived from second analysis phase are presented in results (Table 3).

**TABLE 2:** Summary of schools and participants involved in the study.

	Beginning of academic year	End of academic year	
Data collection	Focus groups (Peer leaders) *n*	Focus groups (Peer leader) *n*	Interviews (ASF coordinators) *n*
Focus groups/interviews	9 (6 online, 3 in‐person)	8 (6 online, 2 in‐person)	6 (online)
Participants	43 (F = 21, M = 22)	33 (F = 22, M = 11)	7 (F = 3, M = 4)
School type			
Mixed gender, DEIS^a^	2	2	2
Mixed gender, non‐DEIS	5	4	2
Single gender, non‐DEIS	2	2	2

Abbreviations: ASF = Active School Flag; DEIS = Delivers Equality of Opportunity in Schools; F = female; M = male.

^a^ (DEIS (Delivers Equality of Opportunity in Schools), a Department of Education and Skills programme in Ireland providing a pathway through education to better opportunities for students in communities at risk of disadvantage and social exclusion).

**TABLE 3:** Themes and sub‐themes for each component of the primary aim, categorized by the extent to which outcomes addressed expectations.

	Extent to which outcomes addressed expectations				
Primary aim	Fulfilled outcomes		Unfulfilled outcomes	Unexpected outcomes	
1 Skills and competencies	Skills for life	Competencies enhanced		Skills for life	Competencies enhanced
	Develop transferable skills Unique skill development opportunities New and existing skills Appreciate relevance of skills Communication Social skills Organization Leadership Teamwork Research Technology Creativity Numeracy	Assertive Maturity Responsibility		Problem solving Time management Adaptable to various roles/tasks	Knowledge of physical activity
2 Health outcomes and behaviors	Physical activity opportunities Boosts confidence Brings mood up Stimulated negative feelings Social connectedness in ASF class Social interaction opportunities			Developed relationships with the whole school community Change in behavior and attitude toward physical activity	
3 School experience	ASF class was a comfortable social/learning environment Enhances school day Positive legacy Role models Challenges encountered		Varied workload Recognition of peer leaders and ASF	Holistic learning experience Developing empathy Long lasting recognition	

*Note:* The themes and sub‐themes are the result of synthesis of data of the preliminary themes and sub‐themes from first analysis phase. Themes/sub‐themes for each component of the primary aim are categorized as to how the peer leaders' outcomes addressed their expectations.

Abbreviation: ASF = Active School Flag.

Legend: 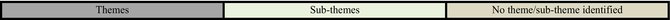

### 3.2 Primary Aim Component 1: Skills and Competencies

Skills and competencies peer leaders expected to develop through ASF were fulfilled. The ASF programme presented additional unanticipated skill development opportunities.

#### 3.2.1 Fulfilled Outcomes

##### 3.2.1.1 Skills for Life

As anticipated, ASF brought peer leaders' skills “to the surface.” Peer leaders developed new and existing skills “transferable to other areas of life” including employment, volunteering, and school roles. Additionally, they appreciated the relevance and importance of the skills.

Peer leaders reported communication skill development including verbal, written, formal communication, public speaking, and listening. Beyond communication social skills were developed through interactions with school members.

Peer leaders felt they enhanced organizational skills when managing logistics, planning, implementing, and reviewing tasks. They used the external ASF survey report to develop their school action plan which involved arranging, supervising, and monitoring WS PA events. After completing tasks, peer leaders reflected on and documented their work in the class ePortfolio. The following describes organizational skills employed:

We organized medals for the first‐year sports day, ringing companies and trying to organize, get money and stuff sorted… great skills to learn (female peer leader, mixed, non‐DEIS school).

These tasks provided opportunities for multiple skill development including professional communication skills to contact companies, logistics planning skills to organize medals, and financial management skills to handle money.

ASF coordinators provided peer leaders with autonomy and trust to lead ASF as one female peer leader (girls only, non‐DEIS school) explained “it was not teachers on top of us twenty‐four‐seven.” Leadership opportunities extended beyond ASF class because peer leaders were perceived as trustworthy. Research skills were developed while sourcing PA information online, which they shared through posters and social media for the ASF “Did You Know?” campaign. Technology skills were enhanced using software and communication tools. Working on WS events helped develop numeracy skills for example, calculating mean step counts.

##### 3.2.1.2 Competencies Enhanced

As expected, assertiveness, maturity, and responsibility were required for their role (e.g., refereeing or coaching PA events). A female ASF coordinator (mixed, DEIS school) perceived that peer leaders' “level of maturity” improved. Peer leaders were given responsibility, autonomy, and independence to work. A male ASF coordinator (boys only, non‐DEIS school) described how peer leaders took initiative “The lads [peer leaders] were like, yeah. ‘Sir, can we grab your keys, we are setting up [the ASF event]’.” Rather than being prompted by the ASF coordinator, the peer leaders themselves initiated contact to complete activities.

#### 3.2.2 Unexpected Outcomes

##### 3.2.2.1 Skills for Life

Overcoming anticipated challenges enabled peer leaders to exceed their skill development expectations. At the beginning of the year, a female peer leader (mixed, DEIS school) doubted their ability to overcome challenges “we don't know if we can take on challenges ourselves.” However, at end of academic year peer leaders reported overcoming challenges. A female peer leader (girls only, non‐DEIS school) cited using problem‐solving, critical thinking, and conflict resolution skills to resolve issues arising “we [peer leaders] have to be the bigger person, and…take charge.” Encountering challenges provided peer leaders with useful but unexpected skills.

Peer leaders anticipated that managing time available to complete tasks would be challenging. A female peer leader (mixed, non‐DEIS school) felt “one class a week can sometimes be a bit short preparing for tournaments.” However, this challenge posed an opportunity to enhance time management skills.

##### 3.2.2.2 Competencies Enhanced

Increased awareness and knowledge of PA was unexpected. A male peer leader (mixed, non‐DEIS) explained when presenting the WS survey results “we would always tell people the recommended amount of activity daily and amount of steps.” Another female peer leader (mixed, non‐DEIS) learned about diverse types of PA “even sports, you wouldn't think of the fencing for example, or you would not think there was an Ireland team for tug of war.” A male ASF coordinator (girls only, non‐DEIS school) witnessed a peer leader becoming aware of the PA guidelines when they said, “I would have classed myself as quite active and I'm still not even hitting the [PA] guideline recommendations.”

### 3.3 Primary Aim Component 2: Perceived Health Outcomes and Behaviors

Peer leaders' expectations were fulfilled in relation to their perception of how their role influenced their physical, social, and mental health. There were two unexpected outcomes and behaviors relating to physical and social health.

#### 3.3.1 Fulfilled Outcomes

##### 3.3.1.1 Physical Activity Opportunities

Peer leaders felt they had PA opportunities as participants, coaches, and referees. A female peer leader (mixed, non‐DEIS school) explained “for other classes we (peer leaders) are just sitting the whole time, but we are doing a lot of stuff outside of the classroom for active school.”

##### 3.3.1.2 Boosts Confidence

As the year progressed, peer leaders experienced positive personal growth reporting feeling confident in themselves and their ability to implement activities. One male ASF coordinator (boys only, non‐DEIS school) acknowledged “some [peer leaders] are very shy to start the year and then toward end of the year they were very confident.”

##### 3.3.1.3 Brings Mood Up

At the beginning of the year peer leaders were excited as they hoped to be in ASF class. This positive mood was maintained throughout the school year as implementing tasks created excitement; a female peer leader (mixed, non‐DEIS school) commented that ASF “brought the mood up.” As hoped, peer leaders were left with positive memories.

##### 3.3.1.4 Stimulated Negative Feelings

As expected, challenges with skills stimulated negative feelings. For example, verbal communication tasks provoked anxiety,

Knowing we (peer leaders) have to talk in front of fifth and sixth years, obviously it's not the worst thing, but you'll be nerve wrecking before (female peer leader, girls only, non‐DEIS school).

##### 3.3.1.5 Social Connectedness in ASF Class

Shared Leadership training enhanced social relatedness among peer leaders,

During the refereeing we split the gym and have one match this side, one match that side and just being able to communicate with people, “I need more people over here to help out” or “I need more people here,” “can someone do this?” … working together for each other (female peer leader, girls only, non‐DEIS school).

In these practical tasks verbal communication skills to request assistance and collaboration skills to complete teamwork were evident.

##### 3.3.1.6 Social Interaction Opportunities

As expected, peer leaders built positive relationships with other adolescents and school management. Networking opportunities occurred with previous peer leaders in their school and with peer leaders in other schools through online meetings to share experiences.

#### 3.3.2 Unexpected Outcomes

##### 3.3.2.1 Developed Relationships with the Whole School Community

Peer leaders expected to “get close to classmates and build relationships” (male peer leader, mixed, DEIS school). Unexpectedly, they enhanced interpersonal relationships with the wider school community, including general student body, staff, and school management.

##### 3.3.2.2 Change in Behavior and Attitude Toward Physical Activity

Peer leaders' became more conscious to do PA. A female peer leader (mixed, non‐DEIS school) described that at home they “try do an hour of being active instead of sitting.”

### 3.4 Primary Aim Component 3: School Experience

Peer leaders school experience fulfilled expectations and there was evidence of unexpected outcomes. Additionally, some expectations of peer leaders' school experience were unfulfilled resulting in unfavorable experiences.

#### 3.4.1 Fulfilled Outcomes

##### 3.4.1.1 ASF Class Was a Comfortable Social and Learning Environment

Peer leaders described ASF as a “standout class” fostering an inclusive and respectful learning environment without pressure or judgment. Shared Leadership training facilitated an equitable and empowering climate as workload was shared. Peer leaders valued ASF class time and the guidance of ASF coordinators. During ASF class peer leaders felt they could express ideas, share opinions, and have their voice acknowledged. One female peer leader (mixed, non‐DEIS school) affirmed that ASF coordinators and school management “listen to us” by acting on their suggestions “they (ASF coordinators and school management) will actually pull through with it (suggestions).”

##### 3.4.1.2 Enhances School Day

At the start of the year peer leaders wanted to be involved in the ASF due to it's positive perception and reputation. The peer leaders' role provided fun and enjoyment as a male peer leader (boys only, non‐DEIS school) outlined without ASF school would be “a lot more plain” and “same day after another.”

##### 3.4.1.3 Positive Legacy

At the end of the year a male peer leader (boys only, non‐DEIS school) felt highly regarded by school members “they (school members) are appreciative of it (ASF) because if we were not doing the (ASF) work, they would not have had opportunity to do all the activities.” Peer leaders felt a sense of purpose and accomplishment as they provided school members with PA opportunities contributing to valuable work in the school.

##### 3.4.1.4 Role Models

As anticipated, an ASF coordinator observed peer leaders as role models for the general student population especially future peer leaders,

They (peer leaders) were role models, and the other younger year groups… particularly the first, second, third (years) and their fellow TY students looked up to them (peer leaders) (male coordinator, boys only, non‐DEIS school).

##### 3.4.1.5 Challenges Encountered

As expected, peer leaders described challenges in ASF due to uncontrollable circumstances (e.g., unfavorable weather). Peer leaders developed resilience when pushed “out of their comfort zone” completing uncomfortable tasks.

#### 3.4.2 Unfulfilled Outcomes

##### 3.4.2.1 Varied Workload

As anticipated, ASF provided peer leaders with a sense of purpose keeping them busy in school. However, in some schools, this was unfulfilled as they completed less tasks. This could be due to a lack of time management as one ASF coordinator acknowledged spending too much time interpreting the ASF survey report, subsequently delaying other task implementation: “I spent less time on (interpreting) the questionnaire (external ASF survey report) than the first year, but I still spent too much time” (male coordinator, mixed, non‐DEIS school). An unintended consequence was ASF having competing school demands resulting in peer leaders missing out on other activities. An ASF coordinator noticed the reluctance of peer leaders to miss lunch break to complete tasks,

We ran one of them (WS events) at lunch, which doesn't seem to go down well, because it's their (peer leaders) lunch and I felt the motivation out of them (peer leaders) wasn't great (female coordinator, mixed, DEIS school).

##### 3.4.2.2 Recognition of Peer Leaders and ASF

Peer leaders hoped ASF would become more visible among school members throughout the year “hopefully we (peer leaders) get noticed after a few events” (male peer leader, mixed, DEIS school). While, school communities were supportive of ASF, peer leaders perceived that ASF remained unrecognized among some school members. Although peer leaders anticipated positive collaborations with teachers in the school, a perceived lack of recognition of ASF among some school members led to this unfulfilled outcome. A female peer leader (mixed, DEIS school) described leaving another module to complete tasks. This was met with lack of understanding of ASF among other module teachers resulting in a negative interaction “sometimes they (non‐ASF teachers) are like, ‘Why are you out of my class, are you avoiding me?’, and we (peer leaders) are like, ‘No, we've [ASF] work to do’.”

#### 3.4.3 Unexpected Outcomes

##### 3.4.3.1 Holistic Learning Experience

Unexpectedly, ASF fostered holistic learning experiences as peer leaders completed practical, team, and independent work providing a welcome “break” from regular school routine.

It is something they (peer leaders) do not do in other subjects. The idea of them (peer leaders) being in charge, going into other lessons, delivering lessons, organizing, liaising with other teachers. It is a very different programme than other things in TY (male coordinator, girls only, non‐DEIS school).

Holistic learning opportunities involved peer teaching through delivery of ASF “Did You Know?” and liaising with teachers providing learning for real world collaboration with adults.

##### 3.4.3.2 Developing Empathy

Unexpectedly, peer leaders became more observant and considerate. The ASF WS survey helped peer leaders gather the wider student voice, and they felt responsible to respond to their peers' requests. Peer leaders considered the ASF coordinators' feedback, ideas, and suggestions and gained a newfound respect and appreciation of authoritative figures “the main things they (peer leaders) learned was how much teachers prepare for a 40‐min lesson” (male coordinator, boys only, non‐DEIS school).

##### 3.4.3.3 Long‐Lasting Recognition

Some coordinators anticipated that peer leaders would continue to be part of ASF on completion of TY, which was not foreseen or noted by peer leaders.

## 4 Discussion

The primary aim was to explore how peer leadership of a WS PA programme influenced adolescents. Peer leadership helped adolescents develop transferable skills, positively influenced their health, and enhanced their school experience. This study highlighted the dynamics of the peer leaders' experiences as some experiences produced ripple effects. Although peer leaders encountered turbulence and challenges, this provided an opportunity for personal growth. Encountering challenges led to the unexpected development of problem‐solving skills. However, in some cases, challenges resulted in negative experiences for peer leaders; for example a perceived lack of recognition for ASF from teachers negatively influenced their school experience. Results will be discussed in relation to SEVT which inform strategies to support adolescent peer leaders' motivation in school‐based interventions.

### 4.1 Fulfilled Outcomes

At the beginning of the year peer leaders expected to develop skills transferable to other aspects of life. Relating to SEVT this indicates the utility value peer leaders had for ASF as they perceived the programme helped develop skills useful to achieve future goals, contributing to positive programme engagement. Skill development came to fruition as peer leaders developed new and existing transferable skills central to teaching and learning across the Irish second level education curriculum [26]. The ASF produced skill development outcomes comparable to outcomes intended to be achieved through training specifically designed for peer leaders to complete in advance of implementing a community sport programme [27], reducing the need for additional resource‐intensive pretraining. This highlights the dual benefit of such peer‐led PA programmes in schools by fostering transferable skills among implementers while providing PA opportunities for the school community. Previous research outlined that peer leaders lacked skills to implement ASF [15]. Rather than adolescents being proficient with skills prior to obtaining the peer leader role, such peer‐led programmes facilitate skill development opportunities organically through implementation.

Physical health was potentially enhanced as peer leaders reported spending less time sitting and had PA opportunities. This was unlike previous PA interventions where peer leaders felt they spent a lot of time sitting and desired intervention components to be more active [22]. Psychologically, peer leadership improved mood and enhanced confidence. However, the role caused transient negative emotions as verbally communicating with school members was a challenge, stimulating anxiety and frustration. Although peer leaders were conscious of possible verbal communication challenges, peer‐led PA programmes should provide further support with communication to ensure a positive experience. Based on SEVT if peer leaders expect that a task is too challenging, they may be less motivated; therefore providing support for challenging tasks could enhance engagement.

In relation to school experience, peer leaders expected ASF would be pleasant. This was evident as the ASF stimulated excitement, demonstrating the intrinsic value peer leaders had for ASF as they perceived the role would be enjoyable, supporting programme engagement. Peer leaders acknowledged the utility value of their work as they felt they contributed to the school community. The ASF class was positively perceived among peer leaders due to its equitable and empowering climate fostered through Shared Leadership training. ASF class was an ideal environment to share their perspectives, with evidence of the Lundy [5] model reflected in the way peer leaders described their experiences. Their accounts illustrate how peer‐led programmes can support adolescents in exercising their right to express their views and ensure they are given fair consideration [28]. Although previous reports suggest TY students felt they did not have a say in the school or classroom [29] this study demonstrated how peer leadership of a WS intervention provided this opportunity. This highlighted the importance of a timetabled class for peer leaders and is consistent with literature [11] that emphasized the importance of infrastructure and policy to support peer leaders achieve programme demands.

Drawing on SEVT, evidence that the ASF programme meets peer leaders' expectations, in addition to indications of its perceived utility and intrinsic value, can strengthen positive beliefs about their capacity to succeed. These outcomes are likely to enhance peer leaders' motivation and engagement; therefore, schools should ensure that peer‐led programmes support and sustain these conditions.

### 4.2 Unexpected Outcomes

Although challenges anticipated by peer leaders at start of the year occurred, these experiences unexpectedly fostered problem‐solving and time management skills. Consistent with the cost component of SEVT, sustaining peer leaders interest and motivation requires that peer‐led programmes ensure the perceived benefits of skills development outweigh the costs associated with encountering such challenges.

An unanticipated school experience was increased consideration of school members and figures in their lives. Empathy development could be due to role modeling, relationship building, teacher support, and student involvement [30]. Adolescents in Ireland may benefit from interventions that support greater consideration of others [30]. Using peer leadership in school‐based programmes may therefore be an effective tool for fostering empathy among adolescents.

These unanticipated outcomes highlight the transformative developmental value of peer leading a school‐based programme, and the added value of such student‐led initiatives. Identifying unexpected outcomes is helpful for determining what further information peer leaders may require to support their engagement. From an SEVT perspective, making peer leaders aware of the potential unexpected benefits may create new value, thereby enhancing motivation and commitment. This finding underscores the importance of unexpected outcomes in shaping task value. Therefore, SEVT could be extended to consider how task value emerges and is constructed through situated experiences, including overcoming challenges.

### 4.3 Unfulfilled Outcomes

Some peer leaders' expectations of their school experience deviated from their actual programme participation. While they hoped that ASF would receive greater recognition from school members, several perceived there was a lack of support and acknowledgement, resulting in unfavorable interactions with their teachers. Teachers may need further information about the programme to be able to provide visible support to peer leaders. As anticipated, there was evidence that the programme kept adolescents busy in school; however, this expectation was unfulfilled for some peer leaders who experienced times with less tasks. Guidance and direction provided by teachers determined the peer leaders' workload, subsequently influencing their school experience.

From an SEVT perspective, unfulfilled outcomes may undermine peer leaders' beliefs in their ability to succeed, possibly reducing their engagement. School‐based, peer‐led programmes should therefore determine whether such outcomes are realistically attainable; if not, it may be useful to communicate this clearly so that peer leaders do not hold unsupported expectations.

### 4.4 Limitations

Only schools at the second stage of ASF were invited to participate, therefore results may not reflect peer leaders' experiences in other programme stages. Convenience sampling by teachers may have resulted in selection based on students' availability, teacher's preference or perceived suitability. Therefore, findings may not fully represent perspectives of the broader student population. Peer leaders' level of engagement in the ASF was not explored which may have influenced reported experiences. Researchers' identities and the focus group format may have influenced the experiences peer leaders felt comfortable sharing. Just over half of the peer leaders that participated at the beginning of the year also participated at the end, therefore findings may overrepresent the experiences of more motivated and engaged peer leaders.

### 4.5 Implications for School Health Policy, Practice, and Equity

The following strategies inform development and implementation of school‐based programmes, peer‐led by adolescents:

- Inform peer leaders that engaging with challenges provides a transformative educational experience. Provide structured opportunities for current and future peer leaders to meet and share experiences of handling challenging situations and highlight how this contributed to skill development.
- School management should formally recognize peer leaders (e.g., in school assemblies) to ensure visible and explicit support.
- School‐based programmes need to resource and support peer leaders when undertaking challenging tasks.
- Aligning such student‐led programmes with curriculum may offer additional benefits for adolescents and facilitate integration into schools.

## 5 Conclusion

Although WS peer‐led PA programmes primarily promote PA, this study highlights their added value in supporting adolescent development among peer leaders. Navigating implementation challenges fostered unexpected personal growth. Findings underscore the benefits and complexities of student‐led initiatives and inform the development and implementation of school‐based, peer‐led interventions.

## Funding

This work was supported by the Healthy Ireland and the Mayo Education Centre.

## Ethics Statement

This study was approved by the University of Limerick research ethics committee (2018_10_18_EHS). ASF coordinators, peer leaders, and their parents/guardians provided informed consent and assent.

## Conflicts of Interest

The authors declare no conflicts of interest.

## Supporting information


**File SA:** Scripts for the focus groups and interviews.

**File SB:** The prelimary themes and sub‐themes from first phase of analysis constructed from peer leader focus groups and coordinator interviews.

## Data Availability

The data that support the findings of this study are available from the corresponding author upon reasonable request.

## References

[1] R. Guthold , G. A. Stevens , L. M. Riley , and F. C. Bull , “Global Trends in Insufficient Physical Activity Among Adolescents: A Pooled Analysis of 298 Population‐Based Surveys With 1.6 Million Participants,” Lancet Child & Adolescent Health 4, no. 1 (2020): 23–35, 10.1016/S2352-4642(19)30323-2. 31761562 PMC6919336

[2] World Health Organisation , Global Action Plan on Physical Activity 2018–2030: More Active People for a Healthier World (World Health Organization, 2018), accessed January 9, 2024, https://iris.who.int/handle/10665/272722.

[3] E. García Bengoechea , C. B. Woods , E. Murtagh , et al., “Rethinking Schools as a Setting for Physical Activity Promotion in the 21st Century–a Position Paper of the Working Group of the 2PASS 4Health Project,” Quest 76, no. 3 (2024): 269–288, 10.1080/00336297.2024.2318772.

[4] Department of Health , Get Ireland Active: National Physical Activity Plan for Ireland (Department of Health Ireland, 2016), accessed December 19, 2023, https://assets.gov.ie/7563/23f51643fd1d4ad7abf529e58c8d8041.pdf.

[5] L. Lundy , “‘Voice’ Is Not Enough: Conceptualising Article 12 of the United Nations Convention on the Rights of the Child,” British Educational Research Journal 33, no. 6 (2007): 927–942, 10.1080/01411920701657033.

[6] K. Fransen , S. A. Haslam , N. K. Steffens , et al., “All for Us and Us for All: Introducing the 5R Shared Leadership Program,” Psychology of Sport and Exercise 51 (2020): 101762, 10.1016/j.psychsport.2020.101762.

[7] A. Carlin , M. H. Murphy , A. Nevill , and A. M. Gallagher , “Effects of a Peer‐Led Walking in ScHools Intervention (The WISH Study) on Physical Activity Levels of Adolescent Girls: A Cluster Randomised Pilot Study,” Trials 19, no. 1 (2018): 31, 10.1186/s13063-017-2415-4. 29325578 PMC5765605

[8] C. L. Edwardson , D. M. Harrington , T. Yates , et al., “A Cluster Randomised Controlled Trial to Investigate the Effectiveness and Cost Effectiveness of the ‘Girls Active’ Intervention: A Study Protocol,” BMC Public Health 15, no. 1 (2015): 526, 10.1186/s12889-015-1886-z. 26036965 PMC4453020

[9] S. J. Sebire , R. Jago , K. Banfield , et al., “Results of a Feasibility Cluster Randomised Controlled Trial of a Peer‐Led School‐Based Intervention to Increase the Physical Activity of Adolescent Girls (PLAN‐A),” International Journal of Behavioral Nutrition and Physical Activity 15, no. 1 (2018): 50, 10.1186/s12966-018-0682-4. 29880048 PMC5992776

[10] M. A. Lavelle , M. Knopp , C. W. Gunther , and L. C. Hopkins , “Youth and Peer Mentor Led Interventions to Improve Biometric‐, Nutrition, Physical Activity, and Psychosocial‐Related Outcomes in Children and Adolescents: A Systematic Review,” Nutrients 15, no. 12 (2023): 12, 10.3390/nu15122658. PMC10300813 37375562

[11] F. McHale , K. Ng , S. Taylor , et al., “A Systematic Literature Review of Peer‐Led Strategies for Promoting Physical Activity Levels of Adolescents,” Health Education & Behavior 49, no. 1 (2022): 41–53, 10.1177/10901981211044988. 34628981 PMC8892039

[12] A. Clerkin , “Personal Development in Secondary Education: The Irish Transition Year,” Education Policy Analysis Archives 20, no. 38 (2012): 1–21, 10.14507/epaa.v20n38.2012.

[13] National Council for Curriculum and Assessment , Transition Year Programme Statement (Department of Education Ireland, 2024), accessed May 9, 2024, https://curriculumonline.ie/getmedia/5849acbf‐0326‐487a‐beec‐3db6b5878470/TY‐Programme‐Statement‐ENG‐INT.pdf.

[14] J. H. Christensen , P. Elsborg , P. S. Melby , G. Nielsen , and P. Bentsen , “A Scoping Review of Peer‐Led Physical Activity Interventions Involving Young People: Theoretical Approaches, Intervention Rationales, and Effects,” Youth & Society 53, no. 5 (2021): 811–840, 10.1177/0044118X20901735.

[15] F. McHale , K. Ng , D. Scanlon , et al., “Implementation Evaluation of an Irish Secondary‐Level Whole School Programme: A Qualitative Inquiry,” Health Promotion International 37, no. 5 (2022): 1–16, 10.1093/heapro/daac131. 36287522

[16] E. Smyth , S. McCoy , and J. Banks , Student, Teacher and Parent Perspectives on Senior Cycle Education (ESRI, 2019), 10.26504/rs94.pdf.

[17] J. S. Eccles , T. F. Addler , R. Futterman , et al., “Expectancies, Values and Academic Behaviors,” in Achievement and Achievement Motives, ed. J. T. Spence (W.H Freeman, 1983), 75–146.

[18] A. Wigfield , “Expectancy‐Value Theory of Achievement Motivation: A Developmental Perspective,” Educational Psychology Review 6, no. 1 (1994): 49–78, 10.1007/BF02209024. 10620382

[19] J. S. Eccles and A. Wigfield , “From Expectancy‐Value Theory to Situated Expectancy‐Value Theory: A Developmental, Social Cognitive, and Sociocultural Perspective on Motivation,” Contemporary Educational Psychology 61, no. 4 (2020): 101859, 10.1016/j.cedpsych.2020.101859.

[20] C. Shang , A. C. Moss , and A. Chen , “The Expectancy‐Value Theory: A Meta‐Analysis of Its Application in Physical Education,” Journal of Sport and Health Science 12, no. 1 (2023): 52–64, 10.1016/j.jshs.2022.01.003. 35051641 PMC9923428

[21] A. Gråstén , W. Anthony , M. Hagger , T. Jaakkola , and J. Liukkonen , “Secondary School Students' Physical Activity Participation Across Physical Education Classes: The Expectancy‐Value Theory Approach,” Physical Educator 72, no. 2 (2015): 340–358.

[22] R. Jago , B. Tibbitts , K. Willis , et al., “Peer‐Led Physical Activity Intervention for Girls Aged 13 to 14 Years: PLAN‐A Cluster RCT,” Public Health Research 10, no. 6 (2022): 1–154, 10.3310/ZJQW2587. 35377571

[23] S. Michie , M. M. van Stralen , and R. West , “The Behaviour Change Wheel: A New Method for Characterising and Designing Behaviour Change Interventions,” Implementation Science 6, no. 1 (2011): 42, 10.1186/1748-5908-6-42. 21513547 PMC3096582

[24] V. Braun and V. Clarke , “Reflecting on Reflexive Thematic Analysis,” Qualitative Research in Sport, Exercise and Health 11, no. 4 (2019): 589–597, 10.1080/2159676X.2019.1628806.

[25] Department of Education and Skills , DEIS Plan 2017 (Department of Education and Skills Ireland, 2017), accessed March 13, 2024, https://www.gov.ie/pdf/?file=https://assets.gov.ie/24451/ba1553e873864a559266d344b4c78660.pdf#page=null.

[26] National Council for Curriculum and Assessment , “Senior Cycle Key Skills Framework,” 2009, https://ncca.ie/media/3380/ks_framework.pdf.

[27] J. H. Christensen , A. B. Evans , C. B. Klinker , M. T. Staal , P. Bentsen , and G. Nielsen , “Activating the ‘Peerness’ of Youth Leaders in a Community Sports Programme Through Techne and Phronesis,” Health Promotion International 37 (2022): 1–11, 10.1093/heapro/daac141. 36300702

[28] United Nations , “Convention on the Rights of the Child. Convention on the Rights of the Child,” 1989, accessed March 1, 2024, https://www.ohchr.org/en/instruments‐mechanisms/instruments/convention‐rights‐child.

[29] Department of Children and Youth Affairs , So How Was School Today? Report of a Survey on How Young People Are Taught and How They Learn (Department of Children and Youth Affairs Ireland, 2017), accessed July 11, 2024, https://www.drugsandalcohol.ie/28126/.

[30] C. Silke , C. Boylan , B. Brady , and P. Dolan , Empathy, Social Values and Civic Behaviour Among Early Adolescents in Ireland: Composite Report (UNESCO Child and Family Research Centre, National University of Ireland Galway, 2019), accessed April 16, 2024, https://www.drugsandalcohol.ie/33339/.

