# Supplementary material for: Personal Development of Adolescent Peer Leaders of a Whole‐of‐School Physical Activity Programme: Expectations Versus Outcomes
Source: J Sch Health. 2026 Sep 10;96(9):e70216. doi: 10.1111/josh.70216 (PMC13561664; doi:10.1111/josh.70216)
Supplement: Supplementary file 1 [file JOSH-96-0-s001.docx]

**Supplementary File A.** Scripts for the focus groups and interviews.

**Peer leader focus group script: Beginning of academic year**

**Icebreaker:** Take a moment to think about how you currently feel about your involvement in the Active Schools Flag class.

**Follow -up:** Tell me why you feel this way in relation to Active School Flag.

1. What do you think the Active School Flag programme is all about?
2. Tell me about the skills you have learned or **hope to learn** while running the Active Schools Flag programme.

**Follow up:** Are these new skills or have you used them before?

3. What things have helped you or will help you run the Active School Flag programme?

**Prompt:** Are there certain skills, people, or Active School Flag resources that has helped/will help you?

4. What challenges have you faced, or do you think you will face when running the Active School Flag programme?

**Prompt:** Challenges you may face could be in the school, personal factors such as skills/personalities etc.

**Follow up:**

- How have/can you overcome these challenges?
- Could you suggest anything that could be done to support your role in the ASF class to help overcome these challenges?

5. In relation to your Active School Flag class tell me about the goals you hope to achieve as a class this year.

**Follow up:** What needs to happen in the class to ensure goals are achieved?

6. From your point of view what do students and staff in the school think about the work you are doing in the Active School Flag class?

**Prompts:**

-Had any praise/compliments from others?

-Has anyone said anything negative to you? What could have been done to make this better?

-Has any of you friends expressed feelings about not being involved in the Active School Flag class?

7. How do you think the principal, vice principal, and board of management in the school feel about the work you are doing to run the Active School Flag programme?

**Follow up:** How do you know that they think/feel this way?

8. Tell me about how you work with your Active School Flag coordinator?

**Prompts:**

-How does this relationship help/hinder your role in the Active School Flag class?

-Is there anything the Active school Flag coordinator could do to make your experience of running Active School Flag better?

-How does ASF class compare to your other TY classes?

9. Do you think that the work you do in Active School Flag class is valuable and important?

**Follow up:** Tell me why you think its important/not important? Who is it valuable and important for?

10. Take a minute to think about the end of the school year when you have completed your time in the Active School Flag T.Y. class. What do you hope to have achieved?

**Prompt:** Personally, for the class, as a leader

**Prompt:** It may be in relation to skills, knowledge, feelings, relationships etc.

**Final question:**

11. Is there anything else you would like to add about Active School Flag that we haven’t already asked?

**Closing:**

Thank you all for taking the time to participate. You input is greatly appreciated.

**Peer leader focus group script: End of academic year**

**Icebreaker:** Take a moment to think about how you currently feel after completing a year in the Active School Flag class?

**Follow up:** Tell me why you feel this way in relation to Active School Flag.

1. Now that you have spent a full academic year in this Active School Flag class can you reflect on this experience and tell me about how you think it has gone?

**Prompt:**

- What were the things you liked about being in this class?
- What are the things you would change about the Active School Flag class? Do you have any suggestions of what could be done to improve your experience of Active School Flag?

**Follow up:** You mentioned at the start of the year that you thought Active School Flag was all about [specify what they previously thought it was about]. After a year of participating in the Active School Flag class, what do you think Active School Flag is all about?

2. Can you describe how implementing Active School Flag programme activities fits into your daily school life.

**Prompt:** This could be organising Active School Flag events, lunch time activities etc.

**Follow up:** Imagine if you did not have Active School Flag in your school, how would this have influenced your TY year?

3. Can you describe a typical class you would have for Active School Flag?

**Follow up:**

- Within the Active School Flag class you had shared leadership roles and responsibilities; how did you find distributing the work this way?
- Tell me about how Active School Flag class compares to your other TY classes?

4. The last time I met with you all, you spoke about the goals you hoped to achieve during your time in the Active School Flag class. You mentioned that you hoped to [specify previous goal mentioned]. Can you update us on how you got on with these goals?

**Follow up:** How did you/what stopped you from achieve your goals?

5. Can you tell me about what has helped you to run the Active Schools Flag programme?

**Follow up:** Can you give me an example of how [help mentioned above] has helped you in the running of the ASF programme?

**Prompt:** Were there certain skills, people or Active Schools Flag resources that has helped you?

6. What challenges have you faced, when running the Active Schools Flag Programme?

**Prompt:** There challenges may have occurred in the Active School Flag class or when running events. Challenges you may face could be in the school, personal factors such as skills/personalities etc.

**Follow up:**

- How did you overcome these challenges?
- Could you suggest anything that could be done to support your role in the Active School Flag class to help overcome these challenges?
- When we met previously you predicted that [specify predicted challenge] could be a challenge. Tell me about how you got on in relation to this predicted challenge? What prevented the challenge/how did you overcome the challenge?

7. Tell me about the skills you have learned or developed while running the Active Schools Flag programme.

**Follow up:**

- Were these new skills or had you used them before?
- Can you give me an example of how you applied/used these skills in Active School Flag during the year.
- When I met you at the start of the year you mentioned that you hoped to develop (specify predicted skills) skills. Tell me about how you got on with developing those skills during the year.

8. From your point of view what do students in the school think about the work you completed in the Active Schools Flag class?

**Prompts:**

- Tell me about any praise/compliments you have heard from students in relation to Active School Flag?  How does this make you feel?
- Tell me about any negative comments you have heard from others in relation to Active School Flag? How does this make you feel? What could have been done to make this better?
- Have any of you friends expressed feelings about not being involved in the Active Schools Flag class?

9. From your point of view what do staff in the school think about the work you completed in the Active School Flag class?

**Prompt:**

- Tell me about any praise/compliments you have heard from teachers in relation to Active School Flag.
- Tell me about any negative comments you have heard for teachers. How did this make you feel?

10. How do you think the principal and vice principal in the school feel about the work you completed for the Active Schools Flag programme?

**Follow up:**

- How do you know that they think/feel this way?
- Can you give an example of any interactions or communication you had with management during your Active School Flag experience.

11. Tell me about how you worked with (name of ASF coordinator teacher) your Active Schools Flag coordinator?

**Prompts:**

- How did this relationship help/hinder your role in the Active Schools Flag class?
- Is there anything the Active schools Flag coordinator could have done to make your experience of running Active Schools Flag better?

**Final question:**

12. Based on your experience of being in the Active School Flag class, if you were to give advice to next year's Active School Flag transition year class, what key things would you tell them?
13. Is there anything else you would like to add about Active Schools Flag that we haven’t already discussed?

If time allows: We are looking to make Active School Flag as good as we can. Is there anything you would improve or suggest doing differently?

**Closing:**

Thank you all for taking the time to participate. You input is greatly appreciated.

**ASF coordinators interview script**

**Ice breaker:** Describe your experience of the Active School Flag programme over the past year in one word.

**Follow up:** Can you expand on why you described your Active School Flag programme experience this year as X?

*For each interview question below I will be asking (i) in general and then (ii) specific to transition year (TY) students in the Active School Flag class.*

1. What aspects of the Active School Flag programme in general, did you like (i) most and (ii) least and why?

**Possible answers/probes**

Describe further… elaborate etc.

Tell me why you liked X the (i) most, (ii) least?

What aspect of Active School Flag did you think was liked most/least by (i) TY students, (ii) teachers, (iii) by the whole school community?

2. How would you describe the collaborative efforts within your school for Active School Flag this year?

**Possible answers/probes**

i) What was support/buy-in like from the teachers?
ii) What was the support/buy-in like from management?
iii) What was buy-in like from the TYs?

If positive, what helped to build support?

If negative, what would you change/do differently?

Drawing other people into the whole-of-school process, is this feasible?

**Follow up:**

Ideally, Active School Flag should be a team effort at manager-teacher-TY level, tell me about your experience of this in your school?

3. On reflection, what do you think **your school got out of the second year of the Active School Flag**?

**Follow up:**

1. How do you think the your second year of the Active School Flag influenced **TY students?** (**Prompt:** when organising Active School Flag events/activities, interacting with other schools, working in the shared leadership groups. **Prompt**: skills and competencies. Get examples of using these skills etc.
2. How do you think being involved in the running of the Active School Flag programme contributes to a TY student's experience of school?

3. What do you think non Active School Flag coordinator **teachers** got out of this stage.

4. Can you describe any specific aspect of the second year of the Active School Flag that (i) you or the (ii) TY students found **challenging** to deliver? (prompt different aspects e.g. shared leadership training, Walkway, organising events).

**Follow up:**

What could have alleviated this/these challenge/s?

How did you overcome the challenge?

5. Since last year has there been any changes in the way you have implemented Active School Flag?

**Prompt:**

1. Structural changes (timetabled class, another coordinator, Active School Flag staff committee)
2. Changes in the way you communicated Active School Flag in school?

**Follow up:** Why were these changes made? What kind of impact did they make i.e. pros/neg?

3. The ePortfolio structure was different this year, how did you find this process? What do you like about the ePortfolios? What would you change about the ePortfolios?
4. How many ASF coordinators are in your school?

**Follow up:**

- **If 2 coordinators**- Tell me about how the workload was shared between you? Did you feel workload was balanced? How did you balance it between you? What could have been done to ensure workload was shared more evenly?
- **If only 1 coordinator**- Why was there only one ASF coordinator involved?

**Follow up:**

How did your experience of Active School Flag, last year influence your ASF (i) delivery (ii) workload?

6. Going forward, what changes would you make to the Active School Flag programme if any?

**Possible answers/probes**

3-year structure (Try-it-out, Cert, Flag), whole-of-school surveys, e-portfolios, shared leadership, resources available, the TY class, the coordinator(s) role etc.

7. What would you describe as your **key** **success** in delivering the Active School Flag programme (i) general and then for the (ii) TY class?

**Possible answers/probes**

What assisted you and the TY class in this/these successes? i.e. People, things, technology, policy?

8. Where does your school go from here with Active School Flag, after your second year participating in the ASF programme?

**Prompts:**

Using the walkway, continuing with Cert stage/moving to flag, implementing physical activity events/extra-curricular physical activity in school

**Follow up:**

Would you lead on Active School Flag in your school again next year?

9. We are now approaching the end of our discussion. Is there anything else anyone would like to add about the Active School Flag programme we have not talked about?
