# Supplementary material for: Personal Development of Adolescent Peer Leaders of a Whole‐of‐School Physical Activity Programme: Expectations Versus Outcomes
Source: J Sch Health. 2026 Sep 10;96(9):e70216. doi: 10.1111/josh.70216 (PMC13561664; doi:10.1111/josh.70216)
Supplement: Supplementary file 2 [file JOSH-96-0-s002.docx]

**Supplementary File B.** The prelimary themes and sub-themes from first phase of analysis constructed from peer leader focus groups and coordinator interviews.

[Insert Figures B1, B2, B3 here]

Figure B1. The first phase of themes and sub-themes from peer leader focus groups at beginning of the academic year.

Figure B2. The first phase of themes and sub-themes from peer leader focus groups at end of academic year.

Figure B3. The first phase of themes and sub-themes from coordinator interviews
